# The iBRA (implant breast reconstruction evaluation) study: protocol for a prospective multi-centre cohort study to inform the feasibility, design and conduct of a pragmatic randomised clinical trial comparing new techniques of implant-based breast reconstruction

**DOI:** 10.1186/s40814-016-0085-8

**Published:** 2016-08-04

**Authors:** Shelley Potter, Elizabeth J. Conroy, Paula R. Williamson, Steven Thrush, Lisa J. Whisker, Joanna M Skillman, Nicola L. P. Barnes, Ramsey I. Cutress, Elizabeth M. Teasdale, Nicola Mills, Senthurun Mylvaganam, Olivier A. Branford, Katherina McEvoy, Abhilash Jain, Matthew D. Gardiner, Jane M. Blazeby, Christopher Holcombe, on behalf of the Breast Reconstruction Research Collaborative

**Affiliations:** 1 Bristol Centre for Surgical Research, School of Social and Community Medicine, University of Bristol, Room 3.12 Canynge Hall, 39 Whatley Road, Bristol, BS8 2PS UK; 2 MRC North West Hub for Trials Methodology Research, Department of Biostatistics, Clinical Trials Research Centre, University of Liverpool, Liverpool, L69 3GS UK; 3 Breast Unit, Worcester Royal Hospital. Charles Hastings Way, Worcester, WR5 1DD UK; 4 Breast Institute, Nottingham University Hospitals NHS Trust, Hucknall Road, Nottingham, NG5 1PB UK; 5 Department of Plastic Surgery, University Hospitals Coventry and Warwickshire NHS Trust, Clifford Bridge Road, Coventry, CV2 2DX UK; 6 The Nightingale Centre Breast Unit, University Hospital of South Manchester NHS Foundation Trust, Southmoor Road, Manchester, M23 9LT UK; 7 Breast Unit, University Hospital Southampton, Tremona Road, Southampton, Hampshire SO16 6YD UK; 8 Faculty of Medicine, University of Southampton, University Road, Southampton, SO17 1BJ UK; 9 Linda McCartney Centre, Royal Liverpool and Broadgreen University Hospital, Prescot Street, Liverpool, L7 8XP UK; 10 New Cross Hospital, Royal Wolverhampton Hospitals NHS Trust, Wednesfield Way, Wolverhampton, WV10 0QP UK; 11 Department of Plastic Surgery, The Royal Marsden NHS Foundation Trust, Fulham Road, London, SW3 6JJ UK; 12 City Hospital, Dudley Road, West Midlands, B18 7QH UK; 13 Imperial College London NHS Trust, London, SW7 2AZ UK; 14 Nuffield Department of Orthopaedics, Rheumatology and Musculoskeletal Sciences, University of Oxford, Nuffield Orthopaedic Centre, Windmill Road, Headington, Oxford OX3 7HE UK

**Keywords:** Feasibility study, Randomised clinical trial, Breast reconstruction, Implant, ADM, Dermal sling, Methodology, Trainee collaboratives

## Abstract

**Background:**

Implant-based breast reconstruction (IBBR) is the most commonly performed reconstructive procedure in the UK. The introduction of techniques to augment the subpectoral pocket has revolutionised the procedure, but there is a lack of high-quality outcome data to describe the safety or effectiveness of these techniques. Randomised controlled trials (RCTs) are the best way of comparing treatments, but surgical RCTs are challenging. The iBRA (implant breast reconstruction evaluation) study aims to determine the feasibility, design and conduct of a pragmatic RCT to examine the effectiveness of approaches to IBBR.

**Methods/design:**

The iBRA study is a trainee-led research collaborative project with four phases:

- Phase 1 – a national practice questionnaire (NPQ) to survey current practice
- Phase 2 – a multi-centre prospective cohort study of patients undergoing IBBR to evaluate the clinical and patient-reported outcomes
- Phase 3– an IBBR-RCT acceptability survey and qualitative work to explore patients’ and surgeons’ views of proposed trial designs and candidate outcomes.
- Phase 4 – phases 1 to 3 will inform the design and conduct of the future RCT

All centres offering IBBR will be encouraged to participate by the breast and plastic surgical professional associations (Association of Breast Surgery and British Association of Plastic Reconstructive and Aesthetic Surgeons).

Data collected will inform the feasibility of undertaking an RCT by defining current practice and exploring issues surrounding recruitment, selection of comparator arms, choice of primary outcome, sample size, selection criteria, trial conduct, methods of data collection and feasibility of using the trainee collaborative model to recruit patients and collect data.

**Discussion:**

The preliminary work undertaken within the iBRA study will determine the feasibility, design and conduct of a definitive RCT in IBBR. It will work with the trainee collaborative to build capacity by creating an infrastructure of research-active breast and plastic surgeons which will facilitate future high-quality research that will ultimately improve outcomes for all women seeking reconstructive surgery.

**Trial registration:**

ISRCTN37664281

## Background

Breast cancer affects 51,000 women each year in the United Kingdom (UK) [1] of whom approximately 40 % [2] will require a mastectomy. The loss of a breast may profoundly impact women’s quality of life [3], and immediate breast reconstruction (BR) is routinely offered to improve outcomes [4].

Implant-based breast reconstruction (IBBR) is the most commonly offered reconstructive procedure in the UK accounting for almost 40 % of all immediate BR performed following mastectomy for breast cancer [5]. Traditional subpectoral IBBR is performed as a two-stage procedure. This is necessary because the subpectoral pocket is too small to accommodate a definitive implant and expansion with a tissue expander is required as a first-stage. Multiple expansions with saline, which may be time-consuming and uncomfortable [6], are then needed before the final implant can be inserted at a second operation.

Over the last 10 years, the introduction of new techniques to augment the subpectoral pocket with biological or synthetic mesh or the patient’s own de-epithelised skin have revolutionised the procedure. The techniques, collectively referred to as lower-pole sling reconstructions (LPSR), may avoid the need for a tissue expander by facilitating single-stage direct-to-implant reconstruction [7] and improve cosmetic outcomes through better lower-pole projection [8–15]. Various products are available and vary widely in cost from £200 for a synthetic mesh such as TiLOOP® [16] or TIGR® Matrix [17] to £1900 for biological alternative [7, 18] such as an acellular dermal matrix (e.g. Strattice® or SurgiMend®). Product selection is currently dependent on surgeon preference.

Despite the rapid adoption of these techniques into routine practice, there have been no well-designed randomised controlled trials (RCTs) comparing them with each other or other BR procedures. One RCT has attempted to compare IBBR with and without a mesh, but closed prematurely due to poor recruitment [19]. Systematic reviews [20–27] have summarised non-randomised studies, but these are at a high risk of bias with significant methodological limitations and cannot be relied upon. A more recent review [28] has critically appraised the evidence and demonstrated a paucity of outcome data relating to the UK practice of single-stage LPSR with non-human biological and synthetic meshes. Comparative data are lacking. Outcomes from UK LPSR studies, however, suggest complication rates may exceed 30 % [29–32] compared the 11 % observed with standard IBBR in the National Mastectomy and Breast Reconstruction Audit [33]. There is therefore a need to robustly evaluate the outcomes of current approaches to IBBR to protect patients and provide evidence for health commissioners to support the provision of these procedures.

Randomised clinical trials provide the best evidence for the effectiveness of most interventions [34], but surgical trials are complex and the challenges to the conduct of well-designed, multi-centre RCTs are well-documented [34–43]. Clinical trials in BR are particularly problematic due to surgeon preference [44–55]. Over the last 20 years, only 14 BR RCTs have been conducted [19, 55–65] of which only four considered IBBR [19, 58, 59, 64]. Systematic reviews [66, 67] have highlighted the need for high-quality BR research but prevailing expert opinion suggests that BR RCTs would be ‘unethical’, ‘impractical’ and/or ‘inappropriate’ [49–53]. Recent qualitative work, however, suggests that well-designed BR RCTs that do not compromise patient choice may be acceptable [68], but identified a lack of understanding of pragmatic trial methodology among BR surgeons as a potential barrier to trial participation [44]. Exploratory, pre-trial work is therefore essential to determine the feasibility of undertaking a trial in IBBR and to inform the optimal study design.

The paucity of data regarding the current practice and outcomes of LPSR in the UK, however, presents particular challenges to the effective design of a pragmatic RCT. There is insufficient data to inform the selection of appropriate comparators, outcomes, sample size or selection criteria. It is also unclear how many patients may be eligible to participate. The need for high-quality data regarding the outcomes of LPSR is increasingly recognised [18, 69], but early progression to a poorly designed trial may alienate potential participants. A large prospective cohort study that engages surgeons, builds collaborations, develops understanding of the need for trials in IBBR and stimulates stakeholder investment in procedure evaluation while generating data to inform the design and conduct of a future trial is therefore necessary before a definitive pragmatic RCT can be initiated.

The National Trainee Research Collaboratives (NTRC) have emerged over the last 5 years as a novel, but effective model for delivery of high-quality multi-centre surgical research [70]. Originating in the West Midlands, the general surgery collaborative network now spans the entire UK [70] alongside subspecialist networks such as plastic surgery [71, 72]. This innovative model of trainees at different hospitals working together has an impressive track record in the design and delivery of large multi-centre cohort studies e.g. the appendicectomy study which recruited 3326 patients from 95 centres over 2 months [73–75] and high-quality surgical trials such as ROSSINI [76] (Reduction in Surgical Site Infection using a Novel Intervention) Trial which randomised 760 patients from 21 centres to a wound protection device versus standard care and recruited ahead of target. Trainees are highly motivated to participate and gain familiarity with research methodology whilst developing practical research skills such as recruiting patients to trials. Trainee networks have the potential to build research capacity and make a significant contribution to clinical research and as such are integral to the new Royal College of Surgeons surgical research infrastructure [70, 77]. This approach has yet to be established in breast and reconstructive surgery but may represent a time and cost-effective means of conducting high-quality research in this area.

## Methods/design

### Aims

The overall aims of the iBRA (implant breast reconstruction evaluation) study are to explore the feasibility, design and conduct of a pragmatic randomised controlled trial (RCT) comparing the effectiveness and cost-effectiveness of novel approaches to implant-based breast reconstruction (IBBR) and the feasibility of using the trainee collaborative model to deliver such a study in breast and reconstructive surgery.

### Study design

The iBRA study will have four phases:

1. Phase 1 will evaluate current practice of implant-based breast reconstruction (IBBR) with a national practice questionnaire (NPQ).
2. Phase 2 will prospectively examine clinical and patient-reported outcomes (PROMs) of IBBR and compare the outcomes of new approaches to surgery with published quality standards [78] and outcomes from the National Mastectomy and Breast Reconstruction Audit (NMBRA) [5, 33, 79, 80].
3. Phase 3 will use mixed methods: an IBBR-RCT acceptability survey (IBBR-RCTAS) developed from phases 1 and 2 and qualitative semi-structured interviews to explore patients’ and surgeons’ attitudes to candidate trial designs and outcomes and their willingness to participate in a future RCT.
4. Phase 4 will incorporate the findings of phases 1 to 3 to inform the design and conduct of a pragmatic definitive RCT with an internal pilot phase which will evaluate the effectiveness and cost-effectiveness of two types of IBBR.

### Phase 1 - National practice questionnaire (NPQ)

The aim of phase 1 is to understand current practice of IBBR in the UK to inform the design of the definitive RCT. This will be achieved with these objectives:

i. To establish the number of centres and surgeons that perform IBBR and are able and willing to participate in a future trial.
ii. To determine the incidence of each type of IBBR being performed at centre and surgeon level (informing trial design and sample size)
iii. To examine how surgeons select suitable patients for surgery (informing the inclusion criteria of the trial)
iv. To determine how the implant procedures are performed at centre level, especially how lower-pole coverage is achieved (informing trial design)
v. To establish current practice of pre and post-surgical care in IBBR which will inform the use and standardisation of concomitant interventions in the trial
vi. To engage surgeons to develop an appreciation of the need for RCTs in IBBR

#### Development of the National Practice Questionnaire

The questionnaire will be developed by members of the steering group. It will ask each centre for information on current IBBR practice (operations and approaches), the multi-disciplinary team in the unit (the type of staff and numbers), the use of novel techniques (dermal slings, biological and synthetic meshes and products used) and how patients are selected for procedure types (the role of neoadjuvant treatments, diabetes, smoking and body habitus). Information about the care pathway in each centre will also be collected.

#### Inclusion criteria for the NPQ

All breast and plastic surgical units performing mastectomy with or without BR in women in the UK will be eligible for inclusion.

#### Data collection in each participating centre

Trainees will be invited by e-mail to participate in the study through the Mammary Fold (MF) Breast Trainees’ Association, the National Trainee Research Collaborative (NTRC) and the Reconstructive Surgery Trials Network (RSTN). Trainees willing to act as the trainee lead for their centre will be asked to contact the study team to express their willingness to participate and to register their Unit. Unit participation and Trainee Lead status will then be confirmed by the study team and relevant documentation sent. Follow-up e-mails will be sent via the MF, RSTN and the professional associations to encourage study participation. Trainee leads will be responsible for identifying a supervising consultant and obtaining the support of other consultants in the Unit. This is the standard approach for NTRC projects which is acceptable and highly effective.

Local Trainee Leads will be responsible for completing the NPQ, paper-based or electronically, with the support of their supervising consultants.

The professional associations (Association of Breast Surgery, ABS and British Association of Plastic, Reconstructive and Aesthetic Surgeons, BAPRAS) have endorsed the study and will encourage Units to participate and support their trainees in recruiting patients and collecting data.

#### Sample size

The National Mastectomy and Breast Reconstruction Audit (NMBRA) [33] collected data from 156 acute Trusts performing mastectomy and BR surgery. Based on a 60 % participation rate, it is anticipated that 100 units may contribute to the study. The study team will approach breast and plastic surgical units via both trainees and the professional associations to maximise participation.

#### Questionnaire data analysis

Questionnaire data will be summarised and summary statistics reported to describe provision of care and practice of IBBR.

Categorical data will be summarised by counts and percentages. Continuous data will be summarised by mean, standard deviation and range if data is normally distributed. Median, interquartile range and range will be reported if the data is skewed. No formal statistical testing will be undertaken.

Where respondents’ free-text responses have been collected these will be anonymised. Thematic analysis will be performed and results presented alongside the quantitative analysis to help contextualise and illuminate them.

#### Feedback and surgeon engagement

Participating units will receive individualised feedback via reports giving their responses alongside cohort averages and national quality standards. This will highlight variation between local and regional practice and enable surgeons and units to compare their local practice to regional and national standards. It will also engage them in the project and highlight the need for a RCT to generate evidence-based practice for regional consistency.

### Phase 2 - Prospective cohort study of patients undergoing implant-based breast reconstruction

The aim of phase 2 is to document the practice and outcomes of IBBR in the UK to inform the design of a definitive RCT. This will be achieved with these objectives:

i. To determine variation in the types of implant-based procedures performed at centre level, in particular, variation in techniques used to achieve lower-pole cover (informing trial design).
ii. To evaluate the practicality of obtaining clinical and patient-reported outcome data across centres with little or no experience in participating in surgical trials, including the feasibility of working with trainees to recruit patients and collect longitudinal data (modelling this on experience in general surgical collaboratives [76]), with a view to using this approach in a future trial.
iii. To compare the outcomes of different approaches of IBBR to published clinical standards and identify risk factors associated with adverse outcomes such as the impact of the learning curve (informing entry criteria, primary and secondary outcomes and parameters required for a power calculation).
iv. To explore the variability of the BREAST-Q between procedure subtypes and hence its value for use in a future trial.
v. To engage and educate consultants and trainees through regular feedback to create a culture of collaborative working and to establish an appreciation of the need for, and a desire to participate in, a future trial in IBBR.
vi. To create the infrastructure to deliver a future trial by developing a network of breast and plastic surgical trainees trained in research methodology supported by consultant surgeons who will participate and recruit patients to surgical trials.

#### Methodology

Centres identified as performing immediate IBBR from the NPQ (phase 1) will be eligible to progress to phase 2, the prospective cohort study. The named supervising consultant will act as the principal investigator for each unit and trainee leads will be responsible for recruitment and data collection.

The study will be piloted in two centres (Liverpool and Bristol) prior to national roll-out to evaluate feasibility and effectiveness of trainee involvement in recruitment and data collection. Methods of data management will also be assessed.

#### Patient inclusion and exclusion criteria

All female patients electing to undergo immediate IBBR for malignancy or risk-reduction under the care of the breast or plastic surgeons will be eligible for inclusion in the study. Excluded will be women undergoing delayed IBBR, revisional surgery and those not able or willing to provide written consent (for patient-reported outcome measures (PROMs) part of the study).

#### Participation identification and recruitment

Potential participants will be identified prospectively by the local study team via clinics, local multi-disciplinary team meetings (MDTs), consultant surgeons and clinical nurse specialists. Simple demographics, procedure and process data will be collected for each participant. Data will be recorded in an anonymised format using a unique alphanumeric study identification number on a secure web-based database. In-hospital complication data will be collected prospectively by trainee leads. Patients will be reviewed in clinics at 30 days to collect complication and oncology data. Note that review will be performed in patients who do not attend for 30 day follow-up.

Trainees or a locally designated member of the team will approach patients in clinics or during their admission to obtain consent for PROMs assessment. Individual centres will be free to determine the optimal approach for recruiting patients to this part of the study. PROMs assessment will be by post or e-mail, dependent on patient preference, at 3 and 18 months (for those eligible for long-term assessment) following surgery and will evaluate satisfaction with care, complications and health-related quality of life (see details below). If consent is obtained, contact details will be sent securely to the co-ordinating centre and questionnaires distributed centrally to optimise compliance, allow accurate follow-up and reduce the incidence of missing data.

#### Sample size considerations

This feasibility study aims to estimate parameters required for an RCT sample size calculation although the primary outcome most suitable is not yet known. Parameters include estimating the standard deviations for each candidate outcome listed below from which, as a result of this study, the most important outcome will be determined and used as the primary outcome when progressing to an RCT.

To inform the assessment of long-term outcomes, 65 patients will be followed-up for a period of 18 months. Allowing for an estimated 20 % loss to follow-up rate (as observed in the National Mastectomy and Breast Reconstruction Audit, NMBRA), long-term data will be collected from approximately 50 patients. This is sufficient to give a reasonable estimate of the standard deviation of the BREAST-Q assessment.

Over the first 12–15 months of the study, the team will aim to recruit as many patients as possible and follow all up to 3 months to estimate with reasonable precision the incidence of each short-term outcome within different treatment approaches. To illustrate, the NMBRA observed an implant loss rate of 9 % in IBBR patients at 3 months. Should this be chosen as the primary outcome in the full trial, establishing this proportion with reasonable precision is vital for the sample size calculation. Using the large sample normal approximation, a sample size of 197 would result in the two-sided 95.0 % confidence interval for a single proportion, assumed to be 0.09, extending from 0.05 to 0.13. Allowing for the 15 % loss to follow-up at 3 months reported in the NMBRA, analysis of implant loss at 3 months would require at least 235 patients to be recruited. Each unit will perform a relatively small number of procedures (4–40 per year) and so the study team will aim to recruit within at least 20 sites.

#### Study outcomes

Currently, it is likely that the primary outcome of the main trial will be based on a clinical effectiveness end-point. This is likely to be one of the outcomes from the recently developed reconstructive breast surgery core outcome set (RBS-COS) [81]. The study management group is currently considering candidate measures below—these will be monitored in phase 2 in terms of data quality to inform the main trial.

- Implant loss: Unplanned and unexpected extirpation or loss of the implant including removal as a result of infection; failure of the reconstruction in the 3 or 18 months following surgery.
- Infection: a hot, red swollen breast associated with one of the following: a temperature, pus at the wound site, a raised white cell count or a positive wound culture in the 3 months following surgery. These are important as they may lead to implant loss and reconstructive failure.
- Major complications: as defined by the RBS-COS: Any adverse event that requires return to the operating theatre or readmission to hospital for any complication related to the reconstruction during the first 3 months following surgery. These include admission for intravenous antibiotics for a wound infection or debridement of skin necrosis and are hypothesised to adversely impact the outcome of the reconstruction.
- Patient satisfaction with the outcome of IBBR as assessed using the BREAST-Q questionnaire at 18 months following surgery.

Additional outcomes of interest from the RBS-COS include quality of life, normality, self-esteem, emotional well-being and physical well-being and will be assessed at 18 months following surgery using the validated BREAST-Q questionnaire [82, 83].

#### Analysis

##### Clinical outcomes

Simple summary statistics will be calculated to describe demographic, procedure, process and outcome data overall and at a site level. Categorical data will be summarised by counts and percentages. Continuous data will be summarised by mean, standard deviation and range if data is normally distributed. Median, interquartile range and range will be reported if the data is skewed. No formal statistical testing will be undertaken.

##### Patient-reported outcome measures – 3 and 18 months

Simple summary statistics will be calculated to describe the parameters identified in the PROMs at 3 and 18 months, and to estimate the variability in outcome in order to determine sample size for the future trial.

Modules from the BREAST-Q questionnaire (Satisfaction with Breast, Satisfaction with Outcome, Psychosocial Well-being; Sexual Well-being; Physical Well-being) will be analysed using the BREAST-Q-specific analysis package and data managed according to BREAST-Q guidelines.

It is anticipated that women undergoing the novel approaches to IBBR will report higher functioning in all domains as a result of having undergone a single versus two-stage procedure with potentially improved cosmetic outcomes. The discriminatory value of the BREAST-Q in detecting differences between procedure subtypes will be explored to determine the benefit of using the BREAST-Q for PROMs assessment in the main trial.

##### Exploratory analysis

A key aim of this study is to determine trial comparators for an RCT. Interventions considered to be prominent are variations in surgical techniques: sub-muscular placement, dermal slings, biological and synthetic meshes. The impact of subtypes of these comparators on the outcomes of interest will be considered.

It is hypothesised that several risk factors may increase the likelihood of an adverse event. Exploratory analysis will help determine best practice to minimise adverse outcomes in the main study.

#### Feedback and surgeon engagement

Individual units will receive anonymised feedback in the form of a report to illustrate variation between local outcomes (clinical and PROMs) and the national standard and outcomes of different procedure subtypes overall. This will be used to highlight variations in the outcomes of IBBR and the need for an RCT to generate evidence-based practice.

### Phase 3 – Using mixed methods to explore the acceptability of proposed trial designs to patients and surgeons

The aim of phase 3 is to use mixed methods (a questionnaire survey and semi-structured qualitative interviews) to investigate the acceptability to patients and surgeons of candidate trial designs generated from phases 1 and 2 to determine a suitable design to progress to a definitive, pragmatic trial. This will be achieved with these objectives:

i. To determine the proportion of patients and surgeons willing and able to participate in each candidate study design
ii. To determine patients’ and surgeons’ views regarding the most appropriate primary outcome for the definitive trial
iii. To determine the degree of pragmatism (e.g. product selection; technique; variation in pre/peri/post-operative care) that would be feasible and acceptable to surgeons in the definitive trial
iv. To explore barriers and facilitators to potential participation in an RCT in IBBR and how these challenges may be overcome.

#### Methodology

##### Randomisation acceptability survey

Using phase 1 and 2 data, two RCT acceptability surveys (RCTAS) (one each for patients and surgeons) will be developed by members of the steering group. Respondents will communicate their willingness to participate in different candidate trials, give opinions regarding primary outcomes and surgeons will offer views about the degree of pragmatism that would be acceptable in a future trial.

#### Participant identification and inclusion criteria

All patients and surgeons who participated in earlier phases will be invited to complete the IBBR-RCTAS by post or online.

#### Analysis

Survey items will be summarised both overall and split by patient and surgeon-respondent groups.

Categorical data will be summarised by counts and percentages. Continuous data will be summarised by mean, standard deviation and range if data is normally distributed. Median, interquartile range and range will be reported if the data is skewed. No formal statistical testing will be undertaken.

Free-text responses will be anonymised and analysed thematically. Findings will be summarised in descriptive accounts supporting conclusions with illustrative quotes. Results will be presented alongside the questionnaire findings to help contextualise and illuminate them.

#### Semi-structured qualitative interviews

Semi-structured qualitative interviews will be used to expand on findings from the questionnaire survey and provide an in-depth exploration of the acceptability of proposed trial designs.

#### Participant identification and sampling

Respondents to the questionnaire who are willing to be contacted will be purposively sampled and interviewed to explore common and unusual questionnaire responses to enable a more detailed understanding of the acceptability of proposed study designs. Patients and surgeons will be interviewed (with the final number depending on data saturation) using a flexible topic guide to investigate their views on willingness to participate in the proposed study designs, appropriate outcome measures and, for surgeons, the degree of pragmatism that would be acceptable and feasible in the definitive trial. Interviews will be audio-recorded, with consent, and fully transcribed.

#### Analysis

Analysis will be an ongoing and iterative process commencing soon after data collection. Transcripts will be imported into the qualitative data analysis software NVivo where they will be systematically assigned codes and analysed thematically to identify key categories using techniques of constant comparison. Preliminary analysis of early interviews will be used to inform further data collection and analysis. A second member of the team, an experienced social scientist (NM), will independently analyse a proportion of transcripts to assess the dependability of coding, and will meet regularly to review coding and descriptive findings, agree further sampling strategies and discuss findings in light of the interview and questionnaire data.

### Data management

Study data will be collected and managed using REDCap electronic data capture tools hosted at University of Edinburgh. REDCap [84] (Research Electronic Data Capture) is a secure, web-based application designed to support data capture for research studies, providing (1) an intuitive interface for validated data entry, (2) audit trails for tracking data manipulation and export procedures, (3) automated export procedures for seamless data downloads to common statistical packages, and (4) procedures for importing data from external sources.

### Criteria for determining progression to main trial design

The feasibility study will determine the most appropriate study design. The primary outcome will be identified, and outcome data used to inform a sample size calculation. Information from rates of surgery and patient selection criteria will be combined with estimates of surgeon and centre willingness to participate in a trial to assess the feasibility of undertaking a definitive trial that would achieve target recruitment within 3 years.

The following specific progression criteria will be reviewed at 30 months:

1. At least 40 % of surgeons and patients responding to the RCT acceptability survey can agree to participate in an RCT in implant-based breast reconstruction surgery
2. The cohort study data and findings of the RCT acceptability surveys (for patients and surgeons) and qualitative work provide sufficient information to inform the selection of an acceptable and appropriate primary outcome measure to inform the sample size required for main study.
3. Based on the sample size calculation for the chosen primary outcome, the number of centres willing to participate, the anticipated patient consent rate, and data from the incident numbers of patients that are likely to be eligible to participate in a RCT, including information about reconstructive techniques used and to be compared in the trial, the study will be practical and achievable with three years of recruitment. Data from an analysis of NIHR portfolio data (Kaur, MRC North West Hub for Trials Methodology Research, personal communication) shows that the trials recruiting to time and target did so with a median recruitment period of 13 months, interquartile range 8 to 24. 42/45 (93 %) recruited successfully within 3 years. Although these data are for paediatric trials, there is no obvious reason why the results would not be generalisable to recruitment in adult studies.
4. Data from the surveys show that a sufficient number of centres, each with a minimum of two participating surgeons are willing and able to participate in the main trial.
5. Feasibility of using the trainee collaborative model to recruit patients and follow them up with <10 % missing data over all the specified time points.

If all points are satisfied, then an application for the main trial using the trainee collaborative model will be made; however, if only points 1–4 are satisfied, then the application will include costings for research nurses to recruit and follow up patients.

It is anticipated that the definitive RCT will compare the effectiveness and cost-effectiveness of two subtypes of single-stage IBBR. It will be large-scale and reflect current practice as determined in the feasibility study. It will be designed with an internal pilot phase which will test whether randomisation is acceptable to patients and surgeons and ensure the processes required for the main trial work together.

### Study governance and consent to participate

Ethical approval is not required for phases 1 and 2 because they involve the routine collection of clinical and patient-reported outcome data as recommended by ‘Oncoplastic Surgery: Guidelines for Good Practice [78]’. Established quality standards for the candidate outcomes have been published [78] and outcomes will be compared to these standards.

Participating centres will therefore be requested to register the study with their clinical audit departments and obtain patient consent for the distribution of patient-reported outcome questionnaires at 3 and 18 months as per previous national studies [79, 85].

Proportional ethics review will be obtained prior to commencing Phase 3, the mixed methods study as this will involve surveying and interviewing patients and professionals regarding the acceptability of a future RCT in IBBR.

## Discussion

The practice of implant-based breast reconstruction has changed dramatically over the last 10 years, but there is a lack of high-quality evidence to support the safety or effectiveness of the new techniques [28]. Randomised trials are needed but the challenges to the successful conduct of surgical trials, and particularly those in reconstructive breast surgery, are well-established [37]. The iBRA study will explore the feasibility of undertaking a large-scale randomised clinical trial in implant-based breast reconstruction. It will use mixed methods to explore the acceptability of candidate comparators and other key trial elements such as primary and secondary outcomes such that the optimal trial design is established. The study will also inform other important design issues including the degree of pragmatism that participating surgeons would find acceptable and determine which concomitant interventions would be permitted within the trial. It is hoped that the iBRA study will lead to the design of a definitive pragmatic multi-centre RCT with an internal pilot phase to explore recruitment issues which will address the clinical and cost-effectiveness of new approaches to IBBR.

If a trial is not found to be feasible, the iBRA study will still be of significant value to patients and professionals as it will represent the largest prospective cohort study of new approaches to IBBR to date. This will provide high-quality observational data to inform decision making for patients and surgeons. The study will also provide a powerful dataset which will allow potential predictors of adverse outcome to be explored. This may inform best practice, lead to the generation of new guidelines and generate hypotheses that will lead to future work in this area. Identification of variations in practice and outcomes may also promote better standardisation of care and improve outcomes for patients considering surgery in the future. Finally, the study will provide a further data cycle following the National Mastectomy and Breast Reconstruction Audit [5, 33, 79, 80] to demonstrate whether outcomes for women undergoing IBBR have improved. If they have not, this will focus the attention of the breast community on relevant areas and highlight the need for future research.

The iBRA study represents the first time that trainee collaborative methodology has been applied to breast and reconstructive surgery and the first time that breast and plastic surgeons have worked together in this way. It is anticipated that the successful conduct of the study will create research capacity by developing a network of trainees and consultants trained in research methodology who will be willing and able to recruit patients to trials and deliver future high-quality research in breast and reconstructive surgery. This will improve the quality and quantity of breast surgical trials and ultimately improve outcomes for patients.

### Study status

Data collection for phase 1 of the iBRA study is now complete with 81 units completing the NPQ. The pilot study was successful and phase 2, the prospective cohort study is currently underway with the first patient recruited to the main study in April 2014. Sixty-five units are currently recruiting to the study and recruitment is ahead of schedule. It is anticipated that recruitment to phase 2 will continue until June 2016.

## Abbreviations

ABS, Association of Breast Surgery; BAPRAS, British Association of Plastic, Reconstructive and Aesthetic Surgeons; BR, breast reconstruction; HES, Hospital Episode Statistics; IBBR, implant-based breast reconstruction; iBRA, implant breast reconstruction evaluation study; LPSR, lower-pole sling reconstruction; MF, Mammary Fold; NMBRA, National Mastectomy and Breast Reconstruction Audit; NTRC, national trainee research collaborative; PROMs, Patient-reported outcome measures; RBS-COS, Reconstructive Breast Surgery Core Outcome Set; RCT, randomised clinical trial; RCTAS, Randomised clinical trial acceptability survey; RSTN, Reconstructive Surgery Trials Network; UK, United Kingdom
